# The experience of seeking recovery interventions for spinal cord injury during the first year: barriers and facilitators

**DOI:** 10.3389/fneur.2025.1541056

**Published:** 2025-05-27

**Authors:** Kim D. Anderson, Anne M. Bryden, Brian K. Gran, Susan W. Hinze, Mary Ann Richmond

**Affiliations:** ^1^Department of Physical Medicine and Rehabilitation, Case Western Reserve University School of Medicine, Cleveland, OH, United States; ^2^MetroHealth Center for Rehabilitation Research, The MetroHealth System, Cleveland, OH, United States; ^3^Department of Sociology, Case Western Reserve University College of Arts and Sciences, Cleveland, OH, United States; ^4^School of Law, Case Western Reserve University, Cleveland, OH, United States; ^5^Jack, Joseph and Morton Mandel School of Applied Social Sciences, Case Western Reserve University, Cleveland, OH, United States; ^6^Spinal Cord Injury/Disorders Center, Veteran Affairs Northeast Ohio Healthcare System, Cleveland, OH, United States

**Keywords:** spinal cord injury, access to care, recovery interventions, clinical trials, support person, barriers to care, rehabilitation, lived experience

## Abstract

**Introduction:**

Spinal cord injury (SCI) is life changing. Recovery is multi-faceted. Knowing that most injuries are incomplete with potential for meaningful recovery and that there is a limited time during which that recovery occurs, maximizing recovery potential early is essential. The objective of this study was to investigate the experience of newly injured people with SCI and their support persons (SP) while they seek out recovery options during the first-year post injury.

**Methods:**

Semi-structured interviews were conducted at three intervals across the first year after having sustained SCI in both Veterans and civilians as well as their SP. Interviews were conducted utilizing an interview guide grounded in two frameworks. Interviews were recorded, transcribed, and deidentified. Codes were developed, revised, or added using a constructivist, grounded theory, analytic approach.

**Results:**

The main source of recovery options was the inpatient rehabilitation team, with delayed access to research teams and people living with SCI. Insurance and institutions are barriers or facilitators to accessing recovery interventions with clear differences between Veteran and civilian healthcare systems. People and knowledge are facilitators. Interest in clinical trials for recovery grows over time, but there are differences based on race. Finding clinical trials and determining eligibility are significant knowledge barriers to the community.

**Discussion:**

This study has revealed knowledge and power imbalances that significantly impede access to recovery interventions sought by people living with SCI and their support persons during their first year after injury. There are clear differences in the experiences of Veterans and civilians.

## 1 Introduction

Spinal cord injury (SCI) is life-changing, producing physical, emotional, and social impacts. Recovery from SCI is multi-faceted, of which physical recovery is one aspect. Most of the physical recovery occurs during the first year of injury (1, 2). Knowing that most injuries are incomplete with potential for meaningful recovery and that there is a limited time frame during which that recovery occurs, maximizing recovery potential through access to various interventions is essential. Acute inpatient rehabilitation immediately after SCI is one important aspect of recovery, but not the only aspect. Evidence indicates that type of insurance often dictates access (3). The average number of days in acute inpatient rehabilitation (lengths of stay) has been declining since the early 1970’s (4). Shortened lengths of stay reduce access to specialized rehabilitative interventions, and do not offer people with SCI opportunities to maximize functional skills because discharge metrics are based on medical stability and not functional independence (5). Shortened lengths of stay also result in increased admissions to skilled nursing facilities, particularly for people with tetraplegia, and skilled nursing facilities provide limited if any therapy (6). Overall, lack of access to acute inpatient rehabilitation as well as shortened lengths of stay when inpatient rehabilitation is accessible, lead to worse outcomes and increased rehospitalizations for secondary complications (4, 5). As such, access to additional recovery options, aside from acute inpatient rehabilitation, that may influence bodily impairment or functional ability is critically important.

More is known about barriers and facilitators to community reintegration (7, 8) than those to recovery interventions. Known patient-specific, provider-specific, and system-related barriers limit access to upper extremity reconstruction surgery interventions for individuals living with tetraplegia (9). Regarding access to assistive technology for individuals with tetraplegia, known barriers are cost, lack of awareness, and eligibility requirements while facilitators are knowledge gained from peers, being connected to resources, and being able to trial-and-error devices (10). There are also known knowledge-related barriers to accessing experimental therapies (11, 12). All of these studies, however, have focused on chronic SCI. Little is known about the experience of seeking recovery interventions early after SCI.

The objective of this study was to use qualitative methods to give voice to the lived experience of newly injured people with SCI (PWS) and their support persons (SP) and to learn about the barriers and facilitators they experience while they attempt to seek recovery and reintegration options during the first-year post injury. The experience was defined by the participants rather than *a priori* assumptions of the research team. The data presented in this manuscript are limited to recovery. Recovery was inclusive of physical and mental health interventions, but exclusive of interventions related to community reintegration. This is because PWS and SP in our study defined recovery primarily in terms of motor, independence, mobility, positivity, emotion, and time whereas reintegration was primarily defined in terms of social roles, participation, and employment (13).

## 2 Materials and methods

All study procedures adhered to the principles of the Declaration of Helsinki. Ethics approval was obtained in late 2019 by the MetroHealth Institutional Review Board (IRB19-00323) and in early 2020 by the VA Northeast Ohio Healthcare System Institutional Review Board (CY19-033).

The data presented here were collected as part of a large qualitative study. A detailed description of the study design, research participants, semi-structured interviews, data collection, and initial data analysis plan has been published (13). In brief, this longitudinal study involved semi-structured interviews that were conducted at three time points (during inpatient rehabilitation, at 6 months post-injury, and at 12 months post-injury) across the first year after having sustained SCI in both Veterans and civilians as well as with their SP. Participant recruitment was via criterion-based (18 years or older, newly acquired spinal damage, participating in initial inpatient rehabilitation stay) purposive sampling and all participants provided informed consent. A sample size of 12–20 per group was targeted as that is the general number needed to reach saturation of emerging themes (14). Interviews with PWS and SP were conducted separately, were in-person or over the telephone, and were done by two interviewers utilizing an interview guide with open-ended questions grounded in the International Classification of Functioning, Disability, and Health (ICF) and Transformative frameworks. The Transformative framework recognizes that knowledge, power, and privilege are important determinants of the realities people experience and that some individuals, including those living with disabilities, are marginalized by social, political, and economic values and the consequent power differentials in healthcare and related institutions (15, 16). Interviews were recorded, transcribed, and deidentified. Using a constructivist, grounded theory, analytic approach, the research team created a codebook based on the interview guide and emergent themes.

### 2.1 Research team

Interviews were conducted by KDA, AMB, BKG, and SWH. All four authors have PhDs, three are female and one is male, two are a professor or associate professor of physical medicine and rehabilitation, and two are a professor or associate professor of sociology. Collectively, the four authors have expertise in conducting qualitative research in SCI, occupational therapy related to SCI, law and social policies, and medical sociology. No relationships were established with participants prior to the start of the study. At the beginning of an interview each interviewer introduced themself and told the participant briefly about their background and interests in the research. KDA, AMB, BKG, and SWH were involved in coding and data analysis. MAR was involved with enrollment and oversight of Veterans and data analysis.

### 2.2 Data analysis

The data presented here results from questions about (1) knowledge of sources and options for recovery, (2) barriers and facilitators experienced while seeking treatments, and (3) attitudes on clinical trials. Trustworthiness was established using multiple strategies. First, the authors who conducted the interviews and coded the transcripts all had requisite expertise, which contributed to credibility. Second, triangulation of multiple data sources all contributed to credibility. One data source was the interview transcripts. Transcripts were first coded independently by four authors, then group review sessions were utilized to reach consensus on codes. During the group review of initial codes, an additional data source was field notes from the interviewers regarding the emotional responses of participants during interviews. Themes and subthemes were derived from the transcripts using a constructivist grounded theory approach (17) until reaching theoretical and substantive saturations (14). The third data source was the demographic and injury-related clinical data collected during the first and third interviews. Finally, member checking was utilized through multiple iterative discussions with our community partner (a local SCI community group) to obtain feedback and perform verification checks of the themes and subthemes.

This project employed a Transformative framework during data analysis as well (15, 16). The Transformative framework recognizes valuable information and evidence study participants possess, experience, and interpret from the moment of their injury throughout the first year of their recovery journey. Because of their diverse backgrounds and circumstances, participants can shed light on structural and institutional challenges and how knowledge and power mitigate or worsen those challenges.

## 3 Results

Demographic characteristics of the enrolled study population have been published as well as information about the population screened (13). In summary, 23 PWS (16 civilian, 7 Veteran) and 21 SP (16 civilian, 5 Veteran) were enrolled. There were some differences between civilian and Veteran PWS including, respectively: 1) 75% vs. 100% male, 2) mean age at injury 41 ± 8 vs. 52 ± 20 years, 3) 44% vs. 14% Black, 4) 50% vs. 86% tetraplegia, 5) inpatient interview at 42 ± 16 days post-injury vs. 79 ± 27 days. All but one SP were female and their social relations to PWS were grandparent (1), parent (5), spouse (7), domestic partner (3), sibling (1), adult child (3), and friend (1). The mean age at enrollment for civilian SP was 47 ± 15 years and for Veteran SP was 53 ± 17 years. Interviews lasted from 30–60 min.

### 3.1 Sources of information about recovery

**Figure 1:**
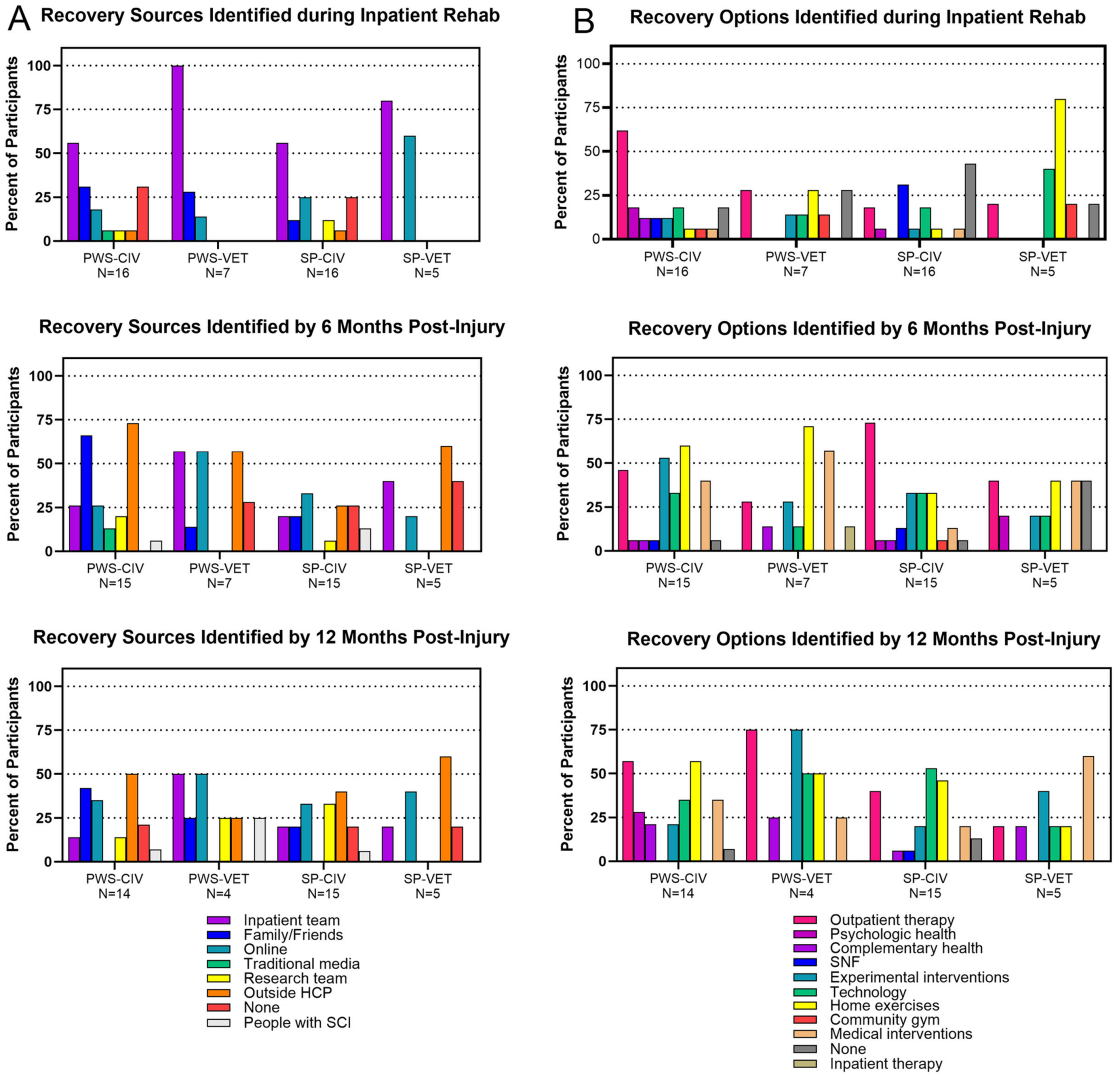
Frequency of themes related to sources and options for recovery. **(A)** Themes regarding recovery sources identified during the inpatient rehabilitation, 6 months post-injury, and 12 months post-injury interviews. **(B)** Themes regarding recovery options identified during the inpatient rehabilitation, 6 months post-injury, and 12 months post-injury interviews. Frequency was based on the number of participants that identified each theme at a particular interview and was categorized by PWS civilian or Veteran and SP civilian or Veteran. Each participant may have discussed more than one theme.

When asked about experiences seeking recovery, participants revealed sources of information for interventions that could impact recovery. In total, 8 themes of sources for information were revealed; they are listed in Table 1 from most to least frequent along with a representative quote. During inpatient rehabilitation the predominant source of information about recovery was the inpatient team, as may be expected (Figure 1A). Veterans’ SP frequently discussed online sources as well. Approximately 25% of civilian PWS or their SP could not identify any sources compared to Veteran PWS or their SP who all identified at least one source of recovery information. At 6 months post-injury (mpi), civilians largely lost access to information from the inpatient team while Veterans did not. Civilians started obtaining information from family and friends as well as outside healthcare providers. There was also the emergence of other PWS becoming sources of information about recovery for civilians. Veterans reported online sources and outside healthcare providers in addition to the inpatient team. There was an emergence of some Veteran PWS or their SP not being able to identify any recovery sources. At 12 mpi, Veteran PWS still discussed the inpatient team and online resources but also began discussing other PWS and research teams as sources of recovery information. Veterans’ SP mostly reported outside healthcare providers and online resources. Civilian PWS began discussing online resources more frequently and their SP started discussing research teams more frequently.

**Table 1:** Themes of sources of information for recovery.

Sources of information for interventions for recovery	
Theme	Representative quotes
Inpatient rehabilitation team	*“Uh, no I have not, but I did have one mention about, um, my hands since I asked about it and they said there was an option for plastic surgery on the hand. Uh, the doctor’s team.”*—C-PWS-14, rehab
Outside healthcare providers (outside of the inpatient team)	*“Um, I I I was referred to her* [researcher in functional electrical stimulation] *by my, um, family physician.”*—C-PWS-20, 6 months
Online	*“Well, I’m, I’ve been looking online and trying to make some phone calls and get a hold of people.”*—C-PWS-20, rehab
Family and friends	*“People give her* [wife] *ideas and give her their phone numbers and she starts calling. So-Friends and people we know. Both. You know, kind of both of them.”*—C-PWS-17, 6 months
None	*“No, no, ain’t nobody came and talked to me about that.”*—C-PWS-21, rehab
Research teams	*“I think she recently sent me the website, which made me go back onto the website and reapply. So actually, Coordinator –Um… She had a little to do with me, uh, going on the SCItrial.”*—C-PWS-12, 12 months
Other people with SCI	*“a friend’s friend, and, uh, who actually had been, uh, consequently shot, and he was, uh, in the wheelchair for about three years.”*—C-PWS-19, 6 months
Traditional media	*“but I’ve seen one on TV actually. It was like a clinical trial for, um, something that they put in the back of our spine.”—*C-PWS-14, 6 months

C = civilian; V = Veteran; PWS = person with SCI; SP = support person; the numbers associated with C, V, PWS, or SP represent different participants.

### 3.2 Options for recovery interventions

Participants also discussed options regarding recovery interventions. In total, 11 themes regarding recovery options were revealed; they are listed in Table 2. During inpatient rehabilitation, outpatient therapy was the primary theme discussed by civilian and Veteran PWS (Figure 1B). Civilian SP primarily discussed skilled nursing facilities or no options for recovery while Veteran SP frequently spoke of home exercise or technology options. By 6 mpi more recovery options were being identified. There was a large increase in civilian and Veteran PWS discussing home exercises and medical interventions. Civilian PWS discussed experimental interventions and technology more often than Veterans. One Veteran discussed the option of returning to inpatient rehabilitation for more therapy, which no civilian mentioned. Civilian SP started discussing outpatient therapy, experimental interventions, technology, and home exercises while Veteran SP also discussed more medical interventions or not knowing any options. At 12 mpi there was an emergence across multiple groups of discussion of psychological and complementary health options for recovery in addition to all the other themes.

**Table 2:** Themes of options of interventions for recovery.

Options for recovery interventions	
Theme	Representative quotes
Outpatient therapy	*“Well, now I’m obviously going to be going to PT and OT still. I have all my appointments set up.”*—C-PWS-12, rehab
Home exercises	*“Well, I do like weights in bed. Like I get in bed, I do weight lifting and I use some, the rubber bands, I use some of them to do, do weight lifting with. I do some with help from my wife-”*—C-PWS-17, 6 months
Technology	*“Of, of that TENS unit. As a matter of fact, uh, I, I, I went ahead and got one.”*—C-PWS-4, 12 months
Experimental interventions	*“The research team, they want to try to rehab me 14 weeks ah, they want to try a couple different things to enhance my recovery.”*—C-PWS-3, 6 months
Medical interventions	*“Uh, well, so I did sign up for a, uh, and I’m waiting for information on a nerve transfer-”*—C-PWS-12, 12 months
None	*“No, nothing I can think of; I have not really gave it much thought, honestly.”*—C-PWS-16, rehab
Skilled Nursing Facilities	*“Um, so when it was time for us to, um, look at nur- … skilled facilities and we were kind of asking, you know, is there one that’s, you know, particularly known, um, for working with spinal cord injuries than the other.”*—C-SP-3, rehab
Psychologic health	*“Now, I’m seeing a psychiatrist. It’s helping a little bit, but…”*—C-PWS-20, 6 months
Complementary health	*“I do look into integrative, um, the Integrative Medical Center at UH. Just looking into that to find out if they did not do hypnosis.”; “Acupuncture, um, uh, meditation-”*—C-PWS-10, 12 months
Community gym	*“Um, so, yeah, and again, the gym, um, that is located near me, I’ll be going to. I’ll be attending that to continue conditioning my strength.”*—C-PWS-12, rehab
Inpatient therapy	*“I’m going back inpatient and basically almost restarting the acute therapy process all over again.”*—V-PWS-15, 6 months

C = civilian; V = Veteran; PWS = person with SCI; SP = support person; the numbers associated with C, V, PWS, or SP represent different participants.

### 3.3 Barriers experienced while seeking recovery interventions

Participants were asked about any barriers they may have encountered while seeking out interventions for their recovery. A total of 9 themes emerged from the transcripts. Supplementary Table 1 contains the name of each theme along with representative quotes.

**Figure 2:**
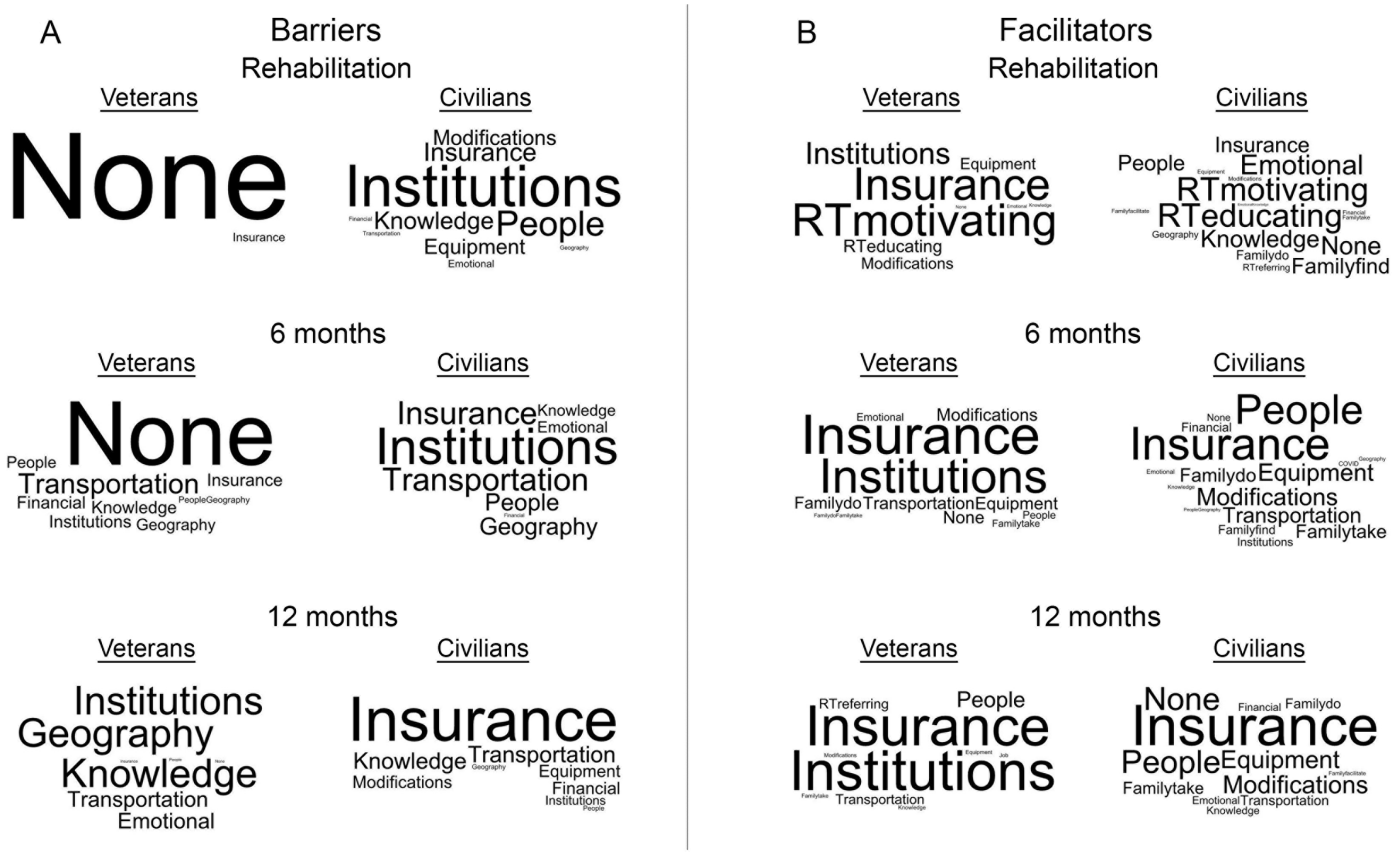
Word clouds representing themes of barriers and facilitators experienced while seeking recovery. **(A)** Barrier themes identified by Veterans and civilians with SCI and their support persons during the inpatient rehabilitation, 6 months post-injury, and 12 months post-injury interviews. **(B)** Facilitator themes identified by Veterans and civilians with SCI and their support persons during the inpatient rehabilitation, 6 months post-injury, and 12 months post-injury interviews. For each word cloud, the size of the text represents the prevalence of which the theme was discussed by participants.

Figure 2A shows word clouds representative of the prevalence of those barriers as civilians and Veterans discussed them during the three interviews and how the barriers change over time. During inpatient rehabilitation, the most prominent barrier theme identified by civilians (PWS and SP combined) related to institutions. This was followed by people, insurance, and knowledge. In stark contrast, almost all Veterans (PWS and SP combined) did not identify any barriers during this period. The one exception was a Veteran that was initially admitted to a civilian hospital and experienced barriers under private insurance. Those barriers resolved once transferred to the VA system. At 6 mpi, civilians still discussed barriers related to institutions and insurance but also experienced an increase in barriers related to transportation, people, and geography. Veterans did start to experience barriers at this time, the most prominent being transportation. Other barriers equally discussed include financial, insurance, people, geography, knowledge, and institutions. At 12 mpi, civilians experienced many more barriers related to insurance followed by knowledge and transportation as well as financial and equipment/modifications. Veterans experienced many more barriers related to institutions, knowledge, and geography followed by transportation and emotions.

### 3.4 Facilitators to accessing recovery interventions

Participants were also asked about any facilitators they may have encountered while seeking interventions for their recovery. A total of 18 themes emerged from the transcripts. Supplementary Table 2 contains the name of each theme along with representative quotes.

Figure 2B contains word clouds representing the prevalence of the facilitators discussed by civilians and Veterans during the three interviews and how the facilitators changed over time. During inpatient rehabilitation, the most prominent facilitators discussed by civilians were members of the rehabilitation team motivating them and educating them about recovery. This was followed by emotions and knowledge. Veterans also discussed the rehabilitation team motivating and educating them as facilitators, but also of strong prominence were insurance, institutions, and equipment/modifications. At 6 mpi, the predominant facilitators discussed by civilians were insurance and people, followed by equipment/modifications, transportation, and family and friends helping them do therapy or taking them to therapy. Veterans, on the other hand, by and far reported their primary facilitators as insurance and institutions followed by much fewer discussions related to transportation, equipment/modifications, and family and friends helping them do therapy. At 12 mpi, the top three facilitators experienced by civilians were insurance, people, and equipment/modifications. Compared to 6 mpi, Veterans discussed facilitators related to institutions and insurance even more prominently at 12 mpi. Other frequently discussed facilitators were people, transportation, and members of the inpatient rehabilitation team referring them to research interventions.

### 3.5 Perspectives on clinical trials

Participants were also asked about their thoughts and interests related to clinical trials and whether they knew how to find trials related to SCI. Interests could be grouped into the three broad categories of Yes, No, or Maybe.

Yes – *“Um, I am 100% for clinical trials. I think that there is nothing better than, uh, getting out there and trying everything I possibly can, uh, to get better. And that’s- that’s only gonna benefit my progression and help others. By doing these clinical trials. And I- I think that maybe that it would be good if anybody really pushed for, uh, outpatient, uh, with these clinical trials, really pushed out that information that there are things such as clinical trials. I had no idea about any of these until I was a couple weeks out of the hospital.”* – V-PWS-11, 6 months.

No – *“Uh, to me I would not want to do it because I, I would rather let somebody else do it before me. Clinical trial means that it is an experiment and they do not know what it is and if it’s going to work or not…Yeah, I feel uncomfortable because I want to know what would, uh, what would happen. What the symptoms of, of, uh, what would happen after I try. It makes me uncomfortable.”* – C-PWS-21, rehab.

Maybe – *“Clinical trials are like a ‘tomato-tomahto’ type situation. It’s not a disagree and it’s not a total agreeance…Because clinical trials are ran and based off of what one theory may be, and what works for one person may not work for another person…So, that’s like a guinea pig-type situation (laughs)…I’m not against it, and I’m not with it, you know? So it’s just like, Uh-… it’s a gray area for me, you know?”* – C-SP-14, 12 months.

**Figure 3:**
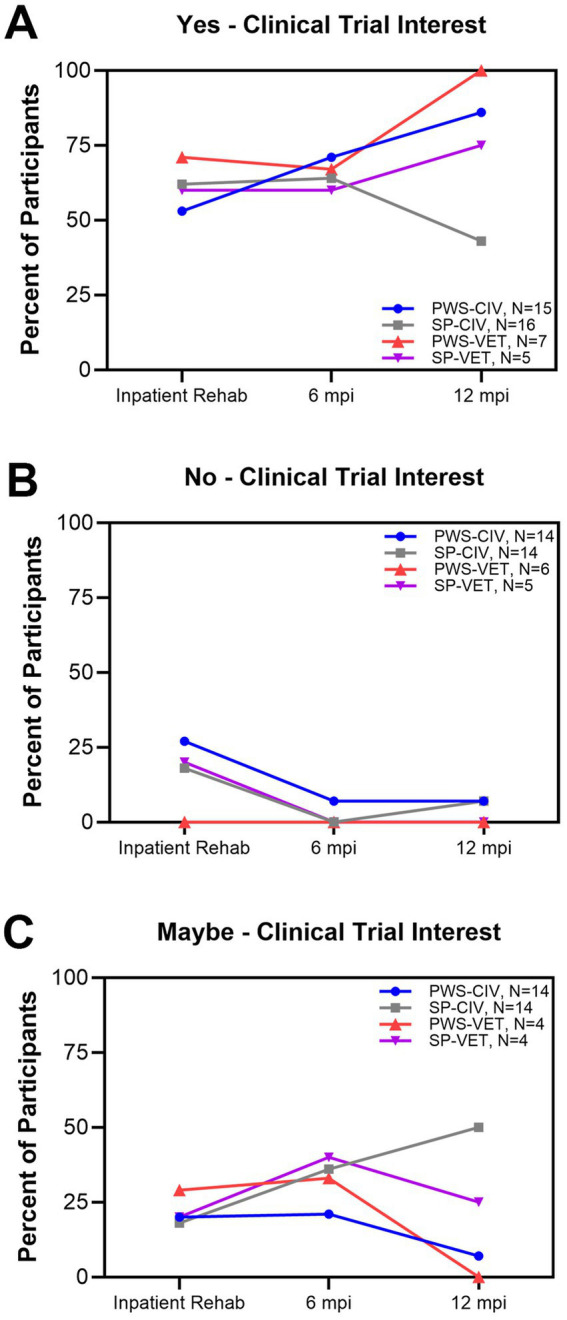
Interest over time in clinical trials as recovery options. **(A)** proportion of participants that indicated that they were interested in trials, **(B)** proportion that were not interested trials, and **(C)** proportion that indicated that they might be interested in trials.

Figure 3 shows the percentage of participants from all four subgroups and the category of interest in clinical trials during inpatient rehabilitation (Figure 3A), at 6 mpi (Figure 3B), and at 12 mpi (Figure 3C). Of note, the proportions of PWS and SP not interested in clinical trials decreased over time. There was a shift from participants not being interested to being possibly or definitely interested in clinical trials as the year progressed. As there is a long history of medical-scientific violations and consequent mistrust in research among different social groups, we analyzed these patterns of interest based on race and identified four patterns. First, during inpatient rehabilitation, there was a stark difference between white and Black participants expressing interest in clinical trials. While 76% of White participants said they were interested, only 31% of Black participants expressed interest. Second, at 6 mpi the percent interested in clinical trials increased in general for both White (86%) and Black participants (44%). However, the gap between groups stayed about the same. Third, at 12 mpi there was a slight increase in interest to 46% of Black participants, and a slight decrease in interest for White participants to 81%. In short, the gap between them declined slightly over time. Fourth, part of the story lies behind those who were not interested in clinical trials over time. For White participants, only 8% expressed no interest during rehabilitation and this dropped to 5% at 6 mpi. For Black participants, the 38% who expressed no interest during rehabilitation dropped dramatically to 0% at 6 mpi. We also analyzed interest in clinical trials based on sex as there is a history of underrepresentation of women in clinical trials. During inpatient rehabilitation, 68% of male participants were interested in clinical trials as opposed to 58% of female participants. At 12 mpi, there was in increase in the proportion of male participants interested in clinical trials (100%) whereas the proportion of female participants interested remained about the same (55%).

Not every participant explicitly stated whether they knew how to find out about SCI clinical trials, but some discussions that emerged from several participants related to the fact that they did not know where to go to find SCI-specific trials.

*“So, I’m open to clinical trials certainly, if I think they would be beneficial to my recovery. But I would, I, I do not know how do you go about finding that.”* – C-PWS-2, 12 months.

Additionally, some participants who had found SCI clinical trials talked about the difficulty of figuring out if they qualified for a trial or not.

*“I am in favor of clinical trials. I think it does something that it should definitely be, um, it should definitely, uh, uh, exist, and I think that it should not be so complicated to figure out, you know, what clinical trial is suitable for me, you know?”* – C-PWS-19, 12 months.

## 4 Discussion

This study reveals detailed insights into the lived experience of seeking recovery during the difficult first year after SCI through the lenses of civilians and Veterans as well as their support persons. Key findings demonstrate a heavy reliance on the inpatient rehabilitation team and delayed access to research teams and people in the community already living with SCI. Insurance and institutions can be critical barriers or facilitators to accessing recovery interventions with clear differences between Veteran and civilian healthcare systems. The fact that no Veterans discussed barriers within the VA healthcare system during inpatient rehabilitation points out the stark differences between a well-funded government/institutional approach to SCI rehabilitation versus a patchwork of privately/publicly funded healthcare services (18). People and knowledge are strong facilitators and have the potential to be leveraged to mitigate some of the barriers that are systemic. Interest in clinical trials as options for recovery grows over time, but there are significant differences based on race and sex. Finding clinical trials and determining eligibility are significant knowledge barriers to the community.

Knowing that the first year of injury is a critical period for maximizing recovery, the delay in finding sources and options for recovery beyond the inpatient rehabilitation team and outpatient therapy highlights the importance of knowledge. This is grounded in the Transformative framework (15, 19) in that knowledge contributes to power and that marginalized groups (i.e., PWS) experience imbalances in power. Level of education, health literacy, and cultural capital are also likely contributors. Our data demonstrate that the inpatient rehabilitation team is a heavily relied upon and influential resource for both civilian and Veteran PWS and SP, but civilians lose access to this source upon discharge. There is an opportunity for inpatient teams to provide knowledge about research while PWS are still undergoing inpatient rehabilitation (e.g., educating about the difference between valid clinical trials and medical tourism), to educate PWS and SP about how to find SCI-specific clinical trials (e.g., www.scitrials.org) and to determine preliminary eligibility, and to connect PWS and SP with research teams in their region. It is important to introduce research as an educational topic during inpatient rehabilitation for a few reasons. One important aspect of education in research is that not all research is cure oriented. There is a large amount of research that is care oriented, such as interventions to impact fitness, prevent secondary complications, and improve self-help strategies. These studies can help people on their recovery and reintegration journey when they no longer have access to traditional rehabilitation. Another important aspect is that the rehabilitation team is a trusted resource. By introducing the concept of research early and providing resources for later use, individuals are armed with this knowledge and can decide to act upon it once out in the community. Facilitating connections with other PWS and SP as additional information sources about recovery is also important to occur early after injury. Our study was conducted during the height of the COVID-19 pandemic when SCI peer mentor resources were restricted from the inpatient setting. This most likely was a strong contributor to our participants experiencing delays in learning that others already living with SCI out in the community are an essential source for recovery information. Gassaway and colleagues (20) demonstrated that intensive peer-mentoring during and after inpatient rehabilitation increased self-efficacy and reduced unplanned hospital admissions in PWS. A scoping review of community-based, peer mentoring interventions suggests that high levels of perceived effectiveness evidence provide support for increased inclusion of such programs in healthcare systems (21). Once out in the community, PWS and SP turn to outside healthcare providers for information about recovery. However, it is known that many healthcare providers do not understand the healthcare needs of people with disabilities in general (22) or SCI specifically (23, 24). Efforts are underway to educate general healthcare providers about disability (25, 26) and SCI (27, 28), but there is much work still to be done.

From our data, we see that insurance and institutions were both barriers and facilitators for different subgroups and at different times during the first year after SCI. For our purposes, we classified discussions related to insurance companies’ coverage of healthcare under the insurance theme and discussions related to policies that impacted access to recovery options, aside from insurance companies’ policies, under the institution theme. The institutional theme included hospital policies, skilled nursing facility policies, government policies, etc. It can be difficult to separate the VA healthcare system from VA institutional policies, but our data clearly show that the two combined are strong facilitators, at least during the inpatient rehabilitation phase, for Veterans with SCI. These provide recovery options such as increased lengths of stay for inpatient rehabilitation, less restricted access to adaptive equipment, coverage for home-based care, and prolonged access to SCI specialists (29). While they did have to navigate VA rules and procedures, Veterans who employed the VA system could count on access to care and rehabilitation. For civilians with SCI, institutional policies are largely barriers to recovery, particularly early after injury. Civilians with SCI in our study experienced insurance as a barrier and facilitator, with insurance becoming more of a facilitator at 6 and 12 mpi. Insurance coverage of the civilian participants was a mixture of private, Medicare, and Medicaid. Participants insured via Medicaid experienced insurance as a facilitator for accessing recovery options over time while those with private insurance or Medicare experienced insurance as a barrier. In fact, two individuals who initially had private insurance switched to Medicaid by 6 mpi and began experiencing fewer barriers while trying to access recovery. Healthcare systems in the United States are complex and different insurance coverage types add additional layers of complexity. The VA healthcare system and insurance coverage is most similar to national health systems and insurance coverage utilized by many international countries. The restriction is that only individuals who have served in the military have access and coverage levels are determined by the degree of service-connectedness. Our data suggests that this kind of system creates minimal barriers during inpatient rehabilitation but does not eliminate all barriers when living outside the hospital setting. Medicaid insurance coverage is at the state level and Medicare coverage is at the federal level. Both could be considered as types of public or social insurance. Medicaid access is generally restricted by income while Medicare is generally restricted by age; however, permanent disability enables eligibility. All other insurance coverage is private. Medicaid, Medicare, and private insurance all independently determine what is covered, how much is covered, and where care can be accessed. Collectively, these restrictions and independent determinations create a bureaucracy of barriers.

The Transformative framework is especially salient in this study by highlighting the participants as experts and the valuable contributions they make to research when communicating their experiences. Results demonstrate how social institutions are layered and overlapping, illuminating power imbalances between people seeking recovery and complex institutional policies and bureaucratic structures that govern access. Some participants face limitations accessing resources. These limitations are intrinsically linked to institutional policies that are often unknown or unclear, or to type of insurance. Understanding how these limitations manifest in people who are marginalized first by sudden onset of disability, and potentially by race, sex, and class, and second by persistent barriers encountered while seeking recovery in society can help identify policies that reduce marginalization, improve access, and ultimately foster recovery.

Sadly, the initial lack of interest in clinical trials by Black participants in our study is not surprising. In general, the data reflect the literature on mistrust in medicine by Black persons who are living “under the shadow of Tuskegee” (30). The history of this abuse has resulted in collective memories for Black persons, contributing to medical distrust, and combined with contemporary negative medical encounters, result in persistent racial disparities in health and healthcare. In their study employing focus groups with Black adults, Scharff (31) found medical mistrust as a primary barrier to participation in clinical trials. Given this history, it is unsurprising that 38% of Black participants in our study expressed *no interest* in clinical trials during rehabilitation. However, by 6 mpi that dropped to 0%. What accounts for the dramatic shift? Perhaps medical mistrust by Black participants in our study declined over time because their interactions with medical providers and staff were unexpectedly positive as they sought recovery. Perhaps talking about clinical trials during our study interviews piqued their interest over time. Over the course of our study, men’s willingness to participate in clinical trials increased whereas women’s willingness was unchanged. Because our PWS were disproportionately male and our SP were disproportionately female, it is difficult to know whether sex is the salient variable. While women have historically been underrepresented in clinical trials, recent emphasis on inclusion has decreased the gender gap. Specifically, Congress passed a 1993 law requiring women to be included in NIH-sponsored trials, increasing gender parity. For example, while only 9% of cardiovascular disease trials included women in 1970, 41% included women by 2006 (32). Clearly, outreach has improved, and newer recruitment strategies have had an impact. In their review of FDA-approved drugs, Labots et al. (33) found that fewer women were included in Phase I trials, but gender parity was reached by Phases II and III. Beyond the biological concern for women’s reproductive health, social factors contribute to lower participation rates. Due to family obligations and a lack of childcare, women may have less free time for participation. As people age, the gender disparities in participation decline, and women might be slightly more likely than men to volunteer. Regardless of race or sex, PWS and SP have difficulty finding information about SCI-specific clinical trials and determining for which studies they might be eligible. It is critical that researchers disseminate their findings on race and sex disparities with respect to clinical trials, engage the multi-cultural community living with SCI to understand their interests and concerns about clinical trials, and incorporate knowledge gleaned from such interactions into co-produced action plans for healthcare institutions, research institutions, regulatory institutions, industry, and community-based organizations to work together to enhance participation in clinical trials that are of interest to PWS and generate knowledge that enhances recovery outcomes.

There are limitations to this study. Most notable is that it was conducted during the COVID-19 pandemic. This impacted participants’ hospital and community experiences. VA hospital restrictions on research during the pandemic also limited our enrollment of Veterans leading us to reach only half of our enrollment target. Additionally, due to the pandemic, most of the interviews were by phone (the first four interviews were just prior to the pandemic and were conducted in person; all others were by phone not using a video platform), which may have led to missed facial expressions and environmental factors that could bring context to discussions. Finally, the data are from a limited geographic region in the Midwest. As such, experiences may be different in different regions, healthcare systems, and social/environmental settings.

## 5 Conclusion

This study has revealed knowledge and power imbalances that significantly impede access to recovery interventions during the first year after injury. This time period is critical for maximizing recovery. There are clear differences in the experiences of Veterans and civilians, particularly related to insurance and institutions. Knowledge and people can help overcome multiple barriers. The inpatient rehabilitation team is highly trusted and has significant knowledge about recovery. Areas to explore include investigating how inpatient rehabilitation teams can connect newly injured individuals with trusted research and community-based knowledge sources, so they do not lose time after discharge trying to find such resources to enhance their recovery. Other areas to explore include determining how research teams at different institutions can help enhance knowledge of and access to clinical trials in a manner that meets the needs and concerns of the multicultural community living with SCI.

## Data Availability

The raw data supporting the conclusions of this article will be made available by the authors, without undue reservation.
