# Supplementary material for: The experience of seeking recovery interventions for spinal cord injury during the first year: barriers and facilitators
Source: Front Neurol. 2025 May 27;16:1541056. doi: 10.3389/fneur.2025.1541056 (PMC12148906; doi:10.3389/fneur.2025.1541056)
Supplement: Supplementary file 1 [file Table_1.docx]

Supplemental Table 1. Barrier Themes Whiles Seeking Interventions for Recovery.

| **Theme** | **Representative Quotes** |
| --- | --- |
| Insurance | *“So, I don't, I don't know how much you know about, if you read, like, all his case notes and stuff, but we weren't even at the rehab, um, building for a week, not even a week, and our insurance company wanted us to leave because they felt there was nothing more they could do for him.”* – Civilian SP (201), rehab  *“Um, I, with therapy I don't, I only get so much therapy a year. So, pretty much every quarter I, quarter, I only get like maybe 10, 10 sessions.”* – Civilian SCI (115), 12 months |
| Institutions | *“So, um,* [son with SCI] *has a felony, um, from ... this is 2020. Maybe three years ago. Um, so I didn't know that facilities, because he has a gunshot wound, was running his record and would not take him based on that. Now, I have been around for 25 years and didn't know that.”* – Civilian SP (205), rehab  *“Oh, and so and I also learned from Social Worker and from the, and from the physical therapist that they cannot recommend, um, or suggest. They can only ... send me a list within 20 miles or 25 miles circumference of* [her] *zip code. And then I had to do, I had to make that decision without any refer, you know, without any reference from* [them*]. I wished, I wish that that team could have given me some input but apparently they're not allowed to”*. – Civilian SP (206), rehab |
| Knowledge | *“I just wish we coulda got here before he got so bad. That's ... But, you know, we just didn't have the knowledge to, you know, come out and tell the doctors, ‘Listen, we need somethin' done.’”* – Civilian SP (204), rehab  *“Um I don't know, I think sometimes it is hard to find stuff and then when you're like researching online and doing that too like, you never know what's really like, legit information I guess and what it isn’t.”* – Civilian SP (216), 12 months |
| People  (not family or friends) | *“Opportunities for recovery. People say they'll get back to us, and don't…The barriers are the people who are supposed to help us but don't seem to be helping right now.”* – Civilian SCI (103), rehab  *“So, I spoke with her about it, and she kind of said, well, they won't accept me* [into a clinical trial] *because I was shot, so my back wasn't broke…Um, she kind of put a damper on my whole situation about that. She really, well, there's pretty much no hope because I had a foreign object in my back, which was, you know, a foreign metal object, and they won't take me because of that.” –* Civilian SCI (102), 6 months |
| Transportation | *“I tried that provide-a-ride, which was a mess. Um, didn’t show up…My little, the robot was calling me, telling me they're gonna be here, you know, so I was up and ready to go an hour and a half before my, my, uh, appointment, out here waiting for them like I'm supposed to be, and they didn't show up.”* – Civilian SCI (115), 6 months  *“Yeah that was, um, that was one of our biggest challenges because- because we make too much money the VA wouldn't come and pick him up for appointments. And our lo- our local VA, um, centers here doesn't have a handicapped accessible van. So we, so we were kind of like caught between a rock and a hard place. So it was, you know, it was a little challenging there in the beginning.”* – Veteran SP (207), 6 months |
| Geography | *“The weather has not real, been more cooperative. That, that, definitely, has, you know ... especially in the h-, in the heat of winter... um, well, actually the cold of winter... getting to* [big city] *has, has kind of cut short some of our, um, therapy trips.”* – Civilian SP (217), 12 months  *“I mean, there were a couple things that are- were somewhat interesting. Um, but just the timeframe just doesn't work out, I guess. And a lot of them were up at the VA. Yeah. That's almost a two-hour drive.”* – Veteran SCI (107), 12 months |
| Equipment/Modifications | *“I feel that that's hindering him right now. So, if we get a couple adaptive equipment changes, and we keep our rehab going with a regular regimen, um, you know, that's our best chance at making more recovery and continuing recovery. Anything less is going to be a regression and a disservice to him based on all the work he's done so far.”* – Civilian SP (201), rehab  *“They're, actually they, they have two things there. Uh, light gate and a standing frame. But they are both too small for me.”* – Civilian SCI (116), 12 months |
| Financial | *“Just like the physical, my primary care doctor said too, most deep muscle massages might help my hands from bending back so far. And they don't... He d-, he d-doesn't do that, but I'm not surprised. Their best Medicare doesn't cover that 80 bucks for a half hour, and I need it frequently. I ca-can't affo-afford it with everything else we want to buy.”* – Civilian SCI (103), 6 months  *“He wants to do physical therapy more, but he doesn't have the money to pay for it out of pocket. And his insurance only covers so many visits per year.”* – Civilian SP (208), 12 months |
| Emotional | *“It is, because he feels like he can't do anything, by not having the use of his limbs.”* – Civilian SP (205), 6 months  *“I think, overall, I mean, uh, the- the resources were there, the information was there, it was the willingness to accept it from us that caused the delay, I'll just say.”* – Veteran SP (207), 12 months |
