# Supplementary material for: The experience of seeking recovery interventions for spinal cord injury during the first year: barriers and facilitators
Source: Front Neurol. 2025 May 27;16:1541056. doi: 10.3389/fneur.2025.1541056 (PMC12148906; doi:10.3389/fneur.2025.1541056)
Supplement: Supplementary file 2 [file Table_2.docx]

Supplemental Table 2. Facilitator Themes Whiles Seeking Interventions for Recovery.

| **Theme** | **Representative Quotes** |
| --- | --- |
| Insurance | *“I have 100% coverage. Um-... from the VA. So all of my medical needs are taken care of. If I pull out the, I think it's called preferred provider-... and they-... are given that they know to bill the VA for whatever medical services a private company would provide. And it allows me to get medical care without worrying of some exorbitant fee coming in.”* – V-PWS-1, rehab  *“Oh, my goodness. I- I ... if we didn't have insurance, I don't know where we would be. I mean, between the VA with all of the medical prescriptions and supplies, and all those things that you need, um, the vehicle, they helped us with the vehicle and the adaptation of the stuff. I- I mean, I- I don't know how people do it if they don't have insurance. I mean, it would be ... it- I mean, we could- I mean, we could do the medicine and stuff like that, but the extras ... we- we definitely would not be able to-The modifications, the- the vehicle would've probably been a struggle. Um, or- or it would've had to have been a plan, then that would probably have taken us just longer to get.”* – V-SP-7, 12 months |
| People  (not family or friends) | *“But, um, I've had an aide here, uh, who is nice enough to, um, work with me, get me range of motion. And, um, um, you know, massage my shoulders, and...my muscles to, to keep them limber and, and whatnot,”* – C-PWS-4, 6 months  *“Um, really just, I mean, anything that I've ever s- ever seen that was, uh, that seemed legitimate was when people would tell me, word of mouth-... to go check out something.”* – V-PWS-15, 12 months |
| Institutions | *“I go back to* [the VA] *on the 13th to finish my therapy and that's gonna be about a month and then I can come back home…Yeah. I'm going back inpatient and basically almost restarting the acute therapy process all over again.”* – V-PWS-8, 6 months  *“I mean, cause everything* [Medicaid-related] *is filtered through* [nursing home]. *We're not really using them independently. Like, it would be a different story when he comes home. You know. Cause then I will have to be the one navigating the Medicaid world. Right now, it's pretty much the facility doing it for us.”* – C-SP-4, 6 months |
| Equipment/Modifications | *“Lightweight things, you know, with, uh, dumbbells. Um, the bands. And I got a, a pulley to put over the door, you know, so he can, he can pull the bands down, he can move about, he can bring them up towards him. You know, all those different things.”* – V-SP-8, rehab  *“Um, well... I guess I would say my new brace, I mean that was really helpful. Yeah, I mean my- my AFO. It's uh, really helped me concentrate on- and getting my knee stronger.”* – C-PWS-23, 6 months |
| Family/friends – do therapy | *“Uh, my wife helps me stretch and move my legs and massages my hands”* – V-PWS-13, 6 months  *“And, that’s that's why I'm trying to work and, uh, like, with my sister to get more, like, to get her to stretch my legs more, like, for longer periods of times”* – C-PWS-12, 6 months |
| Emotional | *“Um... goal, having goals, uh... accomplishing the goal...Every... accomplishing a couple goals every week...Helps mentally overcome the barrier of no progression. So, if I... didn’t get any movement back, or, or any physical strength that... that I used to have, but I crushed all the goals I had for this week, so that's... you know, helpful.”* – V-PWS-11, rehab  *“It's all about your mind. Everything starts within your mind, but if I don't come in this with a great attitude, I will stay sick-... and, and don't heal correctly. If I'm come in with a g- a good attitude and, you know, and just work with the tools that I have-... then I can get through this.”* – C-PWS-5, rehab |
| Transportation | *“if he has any doctors' appointments now, he is using, like, the free, like, um, accessibility, like, transportation vans-... and buses and stuff.”* – C-SP-22, 6 months  *“They [paratransit] are uh, a world of difference. Now-... they're, they're courteous, they're, they're punctual, they're, you know, dependable. You know, it's been so much better. [compared to previous transport agency] So much better. As I call ahead of time, and you know, they do the best they can to get me in, and they get here, they're here. They’re here. [Interviewer: And then what do you usually use them for?] Um, primarily just my, my, therapy appointments.”* – C-PWS-23, 12 months |
| Rehab team – motivating | *“but the trainers for the most part have given me a self-confidence to allow trying new things to, uh, do the distance in the, the hallways here in, uh, the ward.”* – V-PWS-1, rehab  *“Uh, telling me I can do things and, you know, saying that I do have the potential and, you know, motivating me and just giving me things that challenge me and say like, you know, I'm up for the challenge and I can do it. So, you know, just yeah, motivation.”* – C-PWS-14, rehab |
| Family/friends – take to therapy | *“I'm kinda relying on people to drive here and there.”* – V-PWS-11, 6 months  *“I did. At the beginning, I was taking a lot of, um, time off to provide transportation, at this time, I do not have to, um, given that there's other family and friends that stepped in to do the transportation to his doctor's appointment.”* – C-SP-19, 6 months |
| Knowledge | *“What I see for myself is just kind of giving it my all and staying determined and focused because I have done research where people have come back from these, um, type of injuries and it really depends on their commitment towards, um, having, um, perspectives-... the paradigm-... um, about the circumstance and as well as their commitment to, uh, not surrendering themselves to the changes that are happening and occur-... um, in their body, you know? Uh, avoiding states of bitterness, anger, resentment, frustration and, you know, things like that. And I'm not saying those things won't come about, but just try to use strategies to, uh, cope through those, uh, things when they do happen. Stay focused on it.”* – C-PWS-19, rehab  “[Interviewer: Do you feel that your-... experience as a social worker, um, gave you any kind of advantage in trying to sort these issues out-... finding a SNF?] *It-Absolutely.”* – C-SP-3, rehab |
| Rehab team – educating | *“They knew what t-, they knew what the hell they were doing and, and made sure, you know, I learned as much as I could in the short period of time that I was there.”* – C-PWS-12, rehab  *“Yes, yes, I haven’t met a therapist yet that uh... that I wouldn't trust...That wouldn't help me out or educate me on something...They train me.”* – V-PWS-11, rehab |
| Family/friends – find therapy | *“Um, actually, um, my daughter and my girlfriend had been, did a bunch of research, and they, the caseworker there. I forget what her name was, … and then fortunately got referred into this place because I didn't want to go into a hospital that had the COVID-19 because I'm a prime candidate for catching it*.” – C-PWS-20, rehab  *“I got knowledge of* [aquatic therapy center] *through two avenues. One, a friend of mine, and then two, a friend's friend, and, uh, who actually had been, uh, consequently shot, and he was, uh, in the wheelchair for about three years.” –* C-PWS-19, 6 months |
| Financial | *“I received it for 98 days out of the 100 days that Medicare covered. And so I bought the last, I bought from the 28th of August to the 1st of September because I didn't wanna go back home. So I wrote them a check.”* – C-PWS-10, 6 months  *“He has since, um, obtained some equipment at home, like the easystand and-... um, recently, so he can stand a little bit more at home and he's still doing some of the exercises. To make sure that we had the standard, standard frame. Um, and I mean, we just paid for it out of pocket.”* – C-SP-19, 6 months |
| Rehab team – referring to research interventions | *“Uh, Dr. Opal who is his primary doctor in spinal rehab, um, actually submitted my dad's name for like a physical therapy trial of building upper body arm strength up. And dad did the initial interview for that. I don't know how much further it's gone. But that is something he's looking into.”* – V-SP-1, 12 months  *“We would not have known about you or Dr. Khaki,* [researchers] *if Dr. Navy* [rehab team] *would not have guided my husband to you one day when he saw him in the hall, hallway looking befuddled, like where do I turn? So we're so thankful to that man. And guiding us to you guys.”* – C-PWS-2, 12 months |
| Geography | “Um, so, yeah, and again, the gym, um, that is located near me, I'll be going to. I'll be attending that to continue conditioning my strength.” – C-PWS-12, rehab  *“So that's when she had made the suggestion about going into aqua, and, and actually there, there's a sports, um, rehab facility that just opened up through* [location close to home]*, um, and that's how we ended up getting in there.”* – C-SP-18, 6 months |
| Family/friends – facilitate institutional access | *“And the critical care manager there, um, just goes, "We looked into things." And it was* [outside hospital] *and up here at* [civilian rehab hospital]*…Um, some people up here worked... Um, somebody in administration knows her niece. Her niece lobbied hard to get her accepted up here.”* – C-SP-2, rehab  *“Um, well, so, uh, we have three friends, um, that are in- in- in the therapy world. Um, one PT and two, uh, two PTs and one OT. And so in certain instances we have reached out to them for suggestions on, um, um, therapy equipment, recommendations so that he would have the right therapy balls or whatever in- in the gym* [at the nursing home] *when he would go there so he could have his own TENS unit, you know, he helped us with that. Um, and we got our own unit now. … And we thought, well if we give 'em a visual, you know, give 'em illustrations and directions of what we want them to do maybe that would help. So, uh, my one girlfriend who's a PT, sorry OT, gave us a bunch of resources for that.”* – C-SP-4, 12 months |
| Job flexibility | *“Um, my work has been very- ve- very good with me that whenever I need to take off, they- they allow me to, um ...Yeah. I've been very blessed in that- in that aspect, but like it's very challenging. Like I said, we have to be very sche- you know, very deliberate on when we schedule things ... because I try not to be, you know, out all day, every day, but ... 107 probably has, you know, 20 appointments a month in different places ... and we live in a small community, so it's not like it's run down the street. You know, some of these are hour or two hours away. So- so, you know, it just takes coordination and planning, and like I said, I've been ... my work has been very good with me, so I've been able to manage all that, so ...”* – V-SP-7, 12 months |
| COVID-19 | *“Oh, well the pandemic really, um, it real- it for real for real to me it helped me out 'cause I stayed in the house and I can, uh, um, um, work out on my body and everything. So, it did, it did, it did basically, it didn't, it ain't really affecting me because I was already out of commission anyway so, it actually helped me.”* – C-PWS-21, 6 months |

C = civilian; V = Veteran; PWS = person with SCI; SP = support person
